# The Emerging Role of microRNA in Periodontitis: Pathophysiology, Clinical Potential and Future Molecular Perspectives

**DOI:** 10.3390/ijms22115456

**Published:** 2021-05-21

**Authors:** Simona Santonocito, Alessandro Polizzi, Giuseppe Palazzo, Gaetano Isola

**Affiliations:** Department of General Surgery and Surgical-Medical Specialties, School of Dentistry, University of Catania, 95124 Catania, Italy; simonasantonocito.93@gmail.com (S.S.); alexpoli345@gmail.com (A.P.); gpalazzo@unict.it (G.P.)

**Keywords:** microRNA, immune response, periodontitis, exosomes, signalling

## Abstract

During the last few decades, it has been established that messenger ribonucleic acid (mRNA) transcription does not inevitably lead to protein translation, but there are numerous processes involved in post-transcriptional regulation, which is a continuously developing field of research. MicroRNAs (miRNAs) are a group of small non-coding RNAs, which negatively regulate protein expression and are implicated in several physiological and pathological mechanisms. Aberrant expression of miRNAs triggers dysregulation of multiple cellular processes involved in innate and adaptive immune responses. For many years, it was thought that miRNAs acted only within the cell in which they were synthesised, but, recently, they have been found outside cells bound to lipids and proteins, or enclosed in extracellular vesicles, namely exosomes. They can circulate throughout the body, transferring information between cells and altering gene expression in the recipient cells, as they can fuse with and be internalised by the recipient cells. Numerous studies on miRNAs have been conducted in order to identify possible biomarkers that can be used in the diagnosis of periodontal disease. However, as therapeutic agents, single miRNAs can target several genes and influence multiple regulatory networks. The aim of this review was to examine the molecular role of miRNAs and exosomes in the pathophysiology of periodontal disease and to evaluate possible clinical and future implications for a personalised therapeutical approach.

## 1. Introduction

For many years, it was simplistically assumed that biological information was transferred from DNA to messenger RNA (mRNA) and proteins in a linear and sequential manner. This concept was defined as the ‘central dogma of molecular biology’ and was introduced by Crick in 1958 [1]. Today, it is clear that mRNA transcription does not inevitably lead to protein translation, but there are numerous processes involved in post-transcriptional regulation [2], which is a continuously developing field of research. A prominent role in this context is attributed to epigenetic events, which induce changes in gene expression patterns without altering the DNA sequence [3]. These include DNA methylation, histone modification, chromatin remodelling and microRNA (miRNA) interference [4,5]. Epigenetic mechanisms play an important role in chronic inflammatory conditions, as observed in several diseases including cardiovascular disease, Alzheimer’s disease, rheumatoid arthritis and periodontal disease. In periodontal disease in particular, it has been observed that epigenetic alterations promote microbial persistence, creating a microenvironment more favourable to microbial insults. This causes oral pathogens to induce long-lasting interference with the host genome, promoting the onset and development of the disease [6]. 

**Figure 1:**
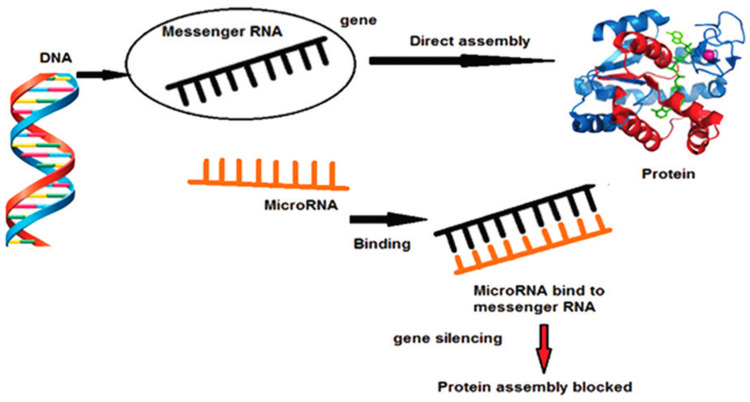
miRNA and inhibiting gene expression changed the concept of the ‘central dogma of molecular biology’ [22].

MiRNAs are short, non-coding, single-stranded RNA sequences that induce post-transcriptional gene silencing. They consist of 18–22 nucleotides, which bind to complementary sequences in the coding or 3’ untranslated region of target messenger RNAs, blocking translation or inducing degradation of target mRNAs [7] (Figure 1). More than 850 mature miRNA sequences have been identified in humans and, although this represents less than 2% of human genes, 30% of mRNAs are predicted to be targeted by miRNAs [8]. Scientific evidence suggests that inhibition of protein synthesis by miRNAs is implicated in several physiological and pathological mechanisms [8]. Indeed, it has been observed that aberrant expression of miRNAs leads to dysregulation of the cellular responses involved in innate and adaptive immune responses, contributing to the development of chronic inflammatory diseases and cancer [9]. Periodontal disease is associated with the host’s immune and inflammatory response induced by pathogenic bacteria, which leads to the destruction of periodontal tissues, with loss of the connective tissue and bone supporting the teeth [10]. The severity and speed of disease progression depend on the dynamic balance of interaction between changes in the oral microbiota and the host’s immune-inflammatory response [11]. The host response to oral bacteria implicated in the development of periodontitis consists of two distinct but interconnected lines of defence: Innate immunity and adaptive immunity [10]. MiRNA regulates the function of neutrophils and their migration from blood capillaries into inflamed tissues by regulating both their adhesion and the stability of chemokine mRNAs [10,12]. It is currently believed that miRNAs may play a role in the pathogenesis of periodontitis, as it has been observed that their dysregulation can be induced by certain bacterial components that are part of the oral bacterial plaque. The action of miRNAs causes the innate and adaptive immune systems to be ineffective in counteracting microbial alteration or to develop an excessive catabolic response [13]. Substantial changes in cellular miRNA levels in diseased tissues compared to healthy ones have been described in several studies. This has been observed in periodontal disease, cardiovascular disease and Alzheimer’s disease [14,15,16]. Therefore, the resulting miRNA profiles have great potential for both diagnostic and prognostic purposes for diseased tissues, including in periodontal disease [13]. 

For many years, it was thought that miRNAs only acted within the cell in which they were synthesised. However, recently, miRNAs have been found outside cells, bound to lipids and proteins or enclosed in extracellular vesicles, namely exosomes. Exosomes are small, 40–130 nm lipid vesicles (bubble-like structures) of endosomal origin used by cells to export material to the space outside the cells [17]. Exosomes containing miRNAs can circulate throughout the body, transferring information between cells and altering gene expression in recipient cells, as they can fuse with and be internalised by recipient cells. Exosomes provide a mechanism for cells to communicate with each other. The critical involvement of exosomes in different types of diseases may clarify potential mechanisms of disease processes [17,18]. Exosomes have been observed to play a role in the regulation of inflammatory, immune and tumour diseases of the oral cavity and beyond, such as rheumatoid arthritis, Sjogren’s syndrome, systemic lupus erythematosus, periodontitis and squamous cell carcinoma of the oral cavity [19]. Therefore, exosomes have captured the interest of scientists, as they could have clinical applications in tissue regeneration, targeted therapy and biomarker research [20,21].

The aim of this review is to examine the role of exosomes and miRNA in periodontitis pathophysiology, and assessments of their clinical potential are discussed in detail.

## 2. MiRNA, Molecular Signalling and Periodontal Disease

For years, bacterial exposure has been regarded as a prerequisite in periodontal inflammation, where microbial biofilm on the enamel surface and in the gingival sulcus induces an immune response of the periodontium [23]. It is now known that bacterial exposure is essential to initiate periodontal disease, but the host response determines the disease phenotype [24]. Epigenetic variations, including miRNAs, and genetic polymorphisms can alter innate or adaptive immune system responses, inducing variations in individual responses, modulation of clinical expression of inflammation and different therapeutic responses. Such genetic and epigenetic influences may classify individuals at high risk of periodontal disease progression [25]. It is known that the host’s periodontal inflammatory response has protective and destructive elements that pathogens can alter. 

Recently, it has been described how *Porphyromonas gingivalis* (*P. gingivalis*), a key pathogen in periodontal disease, manages to evade both innate and adaptive immune responses, facilitating its survival and colonisation within the periodontal pocket [26]. A recent study has shown that the formation of miRNA-584 is induced by *P. gingivalis* lipolysaccharides (LPS). This miRNA, produced by bacterial induction, causes upregulation of IL-8 within gingival epithelial cells by inducing the repression of the lactoferrin receptor, which, if not inhibited, would induce an antimicrobial effect [13,27,28]. 

Host defences generally react to LPS present on the bacterial membrane, involving several protein molecules including tool-receptors (TLRs), nuclear factor-kappaβ (NF-kβ) and so on. The downstream outcome of these activations is the release of cytokines and numerous other pro-inflammatory proteins [13]. Leukocyte migration from the bloodstream into tissues is controlled by the expression of intercellular adhesion molecule-1 (ICAM-1) and E-selectin, which are targets of the miRNA-31 and miRNA-17-3p, respectively [15,29]. MiRNA-31 is downregulated while miR-17 is upregulated in the periodontal tissue of periodontal patients [28]. 

The role of miRNA-146 in periodontitis has been extensively investigated in several studies, because, in response to a bacterial stimulus, it appears to regulate TLR signalling negatively. In fact, once the pathogen has breached the host’s anatomical barriers, TLRs are recognised that activate immune responses [30]. By blocking this signalling process, miRNA 146 appears to favour bacteria’s pathogenic action (including periodontopathic bacteria). Furthermore, according to some studies, miRNA146 is often overexpressed in patients with generalised periodontitis compared to healthy control cases [31,32]. 

Overexpression of this miRNA has been associated with decreasing numbers of cytokines such as TNF-α and IL-1β and NF-κb; therefore, it is thought to prevent excessive tissue damage caused by a disproportionate response [31,33]. However, the literature is not in agreement: in fact, some studies link high miRNA146 levels to the activation of NF-κb, a transcription factor strongly associated with the release of pro-inflammatory molecules, contrary to what was previously described [32]. Therefore, when miRNA146a increases, TNF-α, IL-1β and IL-6 will decrease, inducing a change in the RANK/RANKL/OPG ratio in favour of osteoclastogenesis [34]. This would explain the destruction of alveolar bone tissue induced by the periodontal disease. In agreement with the above findings, Motedayyen et al. found 32.6-fold higher levels of miRNA-146 in tissues with periodontal disease compared to healthy subjects. They also reported a positive association between miRNA146 expression and increased probing depth [34]. Furthermore, the same authors associated MiRNA146a with low levels of EGF (epidermal growth factor) and transforming growth factor-beta (TGFβ) [34]. As both have a primordial role in regulating cell proliferation and survival (especially epithelial), their downregulation, mediated by MiRNA146a, could alter healing processes by promoting therapeutic failures. MiRNA-146a, miRNA-146b and miRNA-155 play a role in regulating TLR release in inflammatory diseases. 

In the study by Xie et al. [15], the expression levels of miRNA-146a and miRNA-146b were found to be significantly higher in inflammatory tissues than in healthy controls. In contrast, miRNA-155 expression levels were significantly reduced in inflamed tissues compared to healthy controls [15]. However, previous studies showed that the expression of miRNA-155 was upregulated in splenocytes, monocytes and macrophages of mice treated with LPS or lipoprotein [35,36,37].

The activity of miRNA-200 also seems to be involved in the pathogenesis of periodontitis. Indeed, in a recent study, its activity in periodontitis was tested, both in vitro and in a rat model, observing that overexpression of miRNA-200c significantly reduced interleukin (IL)-6 and 8 and repressed the interferon-1-related developmental regulator (IFRD1) in primary human gingival fibroblasts (HGF). 

The rat model of periodontitis induced by an injection of LPS into the gingival sulcus of the maxillary second molar (M2) investigated how mediators in the rat gingiva and alveolar bone resorption responded to miRNA-200c treatment by local injection of miRNA-200 PEI-plasmid nanopolyses. Local injection of miRNA-200c significantly increased miRNA-200c expression in the gingiva and reduced IL-6, IL-8, IFRD1 and the ratio of nuclear factor receptor activator kappa-B ligand/osteoprotegerin. This study demonstrated that local treatment with miRNA-200c effectively protected alveolar bone resorption in the rat model of periodontitis, so miRNA-200c could serve as a unique means to prevent periodontitis and associated bone loss [38]. Recently, in a Japanese study, microarrays showed that miRNA-200b levels were increased in inflamed gums and reduced in healthy gums [39]. Furthermore, it was seen that miRNA-200b/c have a regulatory effect on TLR4-mediated signalling in macrophages. This will influence host immune responses to certain periodontal pathogens [40].

MiRNA-21 is upregulated both in patients with periodontitis and in mice induced with periodontitis. In a recent study, the role of miRNA-21 in periodontitis was tested in macrophages challenged by the periodontitis pathogen *P. gingivalis* lipopolysaccharide. MiRNA-21 expression is upregulated in macrophages stimulated by *P. gingivalis* LPS. MiRNA-21 inhibits pro-inflammatory cytokine production by macrophages, whereas miRNA-21 deficiency elevates pro-inflammatory cytokine production. Furthermore, the absence of miRNA-21 promotes the activation of NF-Κb in cells stimulated by *P. gingivalis* LPS. Therefore, miRNA21 has an anti-inflammatory function of miRNA-21 in vitro and in vivo, indicating that miRNA-21 could be an interventional target for the control of periodontitis [41].

Among the miRNAs overexpressed in periodontal tissue affected by disease is miRNA let-7. Its expression increases following exposure to LPS from *Aggregatibacter actinomycetemcomitans* (a typical periodontal pathogen), increasing the inhibition of TLR 4 synthesis and promoting bacterial aggression [42]. A 2018 study by Tiwari et al. hypothesised that miR-let7 family members may play a role in the inhibition of angiogenesis through the upregulation of TSP-1. This would obviously have important pathological repercussions [43]. It has been shown that angiogenesis plays an essential role in maintaining periodontal homeostasis. It is regulated by several growth factors and cytokines, including basic fibroblast growth factor (b-FGF), endoglin, platelet and endothelial cell adhesion molecules (PECAM-1), vascular endothelial growth factor (VEGF), soluble intercellular adhesion molecule-1 (sICAM-1) and soluble vascular cell adhesion molecule-1 (sVCAM-1) [44]. MiRNA-let-7 is upregulated in the inflamed periodontal tissue of moderate-to-advanced periodontitis patients [45]. 

In another study conducted in India in 2017, miRNA samples obtained from 100 subjects with periodontitis and 100 periodontally healthy subjects were compared. Results showed increased expression of miRNA-let7a and miRNA-21 in periodontopathic subjects. Downregulated miRNAs included miRNA-125b and miRNA-100, which showed a 1.6-fold reduction in periodontitis patients compared to healthy controls [46]. The levels of miRNA-203 were also found to be reduced compared to healthy tissue, an observation that fully agrees with their known anti-pluror function [28]. Interestingly, miRNA-203 activity was correlated with keratinocytes, where it is an important regulator of wound-specific cellular functions and the cytokine network associated with wound healing [10]. The lower expression of miR-203 in periodontitis is consistent with increased angiogenesis in periodontitis. In contrast, when its expression levels are normal, it targets vascular endothelial growth factor alpha (VEGFA), inhibiting angiogenesis [8]. These findings would suggest a protective role of miR-203 in chronic periodontitis and its potential to be used as a healing-promoting therapeutic target.

MiRNA103, miRNA22 and miRNA106b have target genes related to inflammation and bone metabolism (IL, PGE2, TNF, etc.). These miRNAs were found to be upregulated in tissues with periodontal disease [28,47]. Another similar study by Xie et al. from 2011 [15] shows that there is a two-fold increase in 91 miRNAs in periodontopathic gum sites compared to healthy gums and a decrease in another 34 miRNAs. Twelve of the 91 upregulated miRNAs, including miR-126-5p, miR-20a, miR-142-3p, miR-19a, let-7f, miR-203, miR-17, miR-223, miR-146a, miR-146b, miR-155 and miR-205, are associated with inflammatory processes, as reported by TargetScan and miRNA.org—databases storing all miRNA data [15].

Luan et al. found that miRNAs upregulated due to periodontal disease in both humans and mice included miRNA-15a, miRNA-29b, miRNA-125a, miRNA-146a, miRNA-148/148a and miRNA-223, while miRNA-92 was downregulated [10]. It has also been found that there are no major qualitative differences in miRNA expression in chronic or aggressive periodontitis [48]. Different microarray studies have demonstrated the over-regulation of miRNA-223 in gingival tissue biopsies from patients with periodontitis compared to healthy gingiva [15,28,39]. Furthermore, Tomofuji et al. confirmed that miRNA-223 was one of the miRNAs overexpressed in the serum of a periodontitis mouse model. miRNA-223 works as a principal regulator of innate immunity and, under normal conditions, participates in tissue homeostasis [49]. It has been found to be dysregulated in many inflammation-related disorders. miRNA-223 is also involved in the differentiation of several immune cells, particularly macrophages, by influencing their activation patterns [50]. Therefore, miRNA-223 plays an essential role in the early stages of infection and inflammation. Periodontitis is characterised by an exaggerated immune response to periodontal pathogens [51], where neutrophils are highly predominant and their hyperactivation is thought to be induced by miRNA-223 [52]. Numerous studies have documented the potential function of miRNA-223 expression in controlling osteoblast differentiation, where its involvement, along with miRNA-214 and miRNA-338, suppressed osteogenesis by supporting osteoblast apoptosis and stimulating osteoclast differentiation by targeting nuclear factor 1-A (NF1-A). Therefore, increased levels of miRNA-223 in inflamed gingival tissue play a role in alveolar bone loss, which is an emblem of periodontitis [53].

A summary of miRNAs relevant to the pathogenesis of the periodontal disease is given in Table 1.

Therefore, the expression of some miRNAs is associated with *P. gingivalis* itself [55], while the expression of others, such as miRNA-128, miRNA-146, miRNA-203 and miRNA584, is derived from the host’s response to infection. Interestingly, *P. gingivalis*-associated miRNAs can influence the host’s innate immune responses (directed against it), whereas its LPS can alter and reduce miRNA–mRNA interactions of the infected organism. These miRNA-dependent effects may complement other forms of deception exerted by *P. gingivalis* to attempt to subvert the host’s innate and adaptive immune responses [13].

## 3. MiRNA as a Possible Link between Periodontal Disease and Various Systemic Disorders

The role of the oral microbiota and its immune subversion strategies in supporting local and systemic inflammation is well established [56]. The various deleterious effects of periodontitis include bacteraemia, endotoxemia and low-grade systemic inflammation, which have been associated with systemic diseases such as diabetes mellitus and coronary artery disease [57]. Indeed, a disproportionate hyperinflammatory response leads to chronic non-resolving inflammation in which epigenetic factors, oxidative stress and cytokines play an important role [52].

The literature now recognises the link between cardiovascular disease and periodontal disease [58,59]. However, the pathophysiological links between these two diseases are still not entirely clear. MiRNAs, as seen above, are currently implicated in the regulation of numerous cellular processes, including inflammatory disorders, and can be considered as the key link between cardiac and periodontal disorders. However, the literature on this subject is sparse, with very few recent studies. Considerable interest has been expressed in a possible correlation between miRNA146, periodontal disease and cardiovascular disorders [36]. The release of miRNA-146a leads to a reduction in adaptor proteins involved in inflammatory responses such as TRAF6 and IRAK1 and their associated pro-inflammatory cytokines. This reduction will reduce the NF-κB cascade, which leads to the formation of pro-inflammatory cytokines [36]. Thus, miRNA-146a paradoxically acts as a negative regulator of the inflammatory process [36]. Despite these properties, miRNA-146a has been associated with disorders with inflammatory pathogenesis, such as atherosclerosis, diabetes and periodontitis [30,60]. In a recent case–control study, Bagavad et al. analysed and studied mir-146a in subjects with acute coronary syndrome (ACS) and in patients with and without chronic periodontal disease (CP). Patients were divided into four groups, each consisting of 66 patients: group I with patients with ACS but without CP, group II with patients with ACS and CP, group III containing patients with only periodontitis and, finally, a control group of cardiac and periodontally healthy subjects. The results suggested that miR-146a was often significantly associated with subjects in group II (patients with acute coronary syndrome and periodontal disease) [30]. The study also found that circulating miR-146a levels were upregulated in all groups of diseased subjects (groups I, II and III) and downregulated in the healthy control group (group IV) [30]. The same study hypothesised that these small ribonucleic acids could lead to the onset of inflammatory disorders in both the heart and periodontal regions by acting against th1/th2 cells and shifting the balance towards the th1 group [30]. In more recent study, miRNA-146a levels were quantified in subgingival plaque samples and then correlated with periodontal and cardiac parameters in patients with chronic periodontitis (CP) with and without coronary heart disease (CHD) [61]. The periodontal parameters examined were plaque index, bleeding on probing, pocket depth on probing and clinical attachment levels, while the cardiac parameters taken into account were total cholesterol, high-density lipoprotein, low-density lipoprotein, triglyceride levels and blood pressure (systolic and diastolic). Higher miRNA-146a levels were found in the CP + CHD group and a positive correlation was shown with index body mass and periodontal and cardiac parameters. Thus, it was further confirmed that miR-NA-146a is involved in the pathogenesis of both periodontitis and coronary heart disease [61].

A bidirectional relationship between diabetes and periodontitis has been demonstrated for years. In fact, it has emerged that periodontitis is a very common complication in patients with diabetes. In particular, periodontitis has emerged as the ’sixth complication’ of diabetes, after retinopathy, neuropathy, nephropathy, macrovascular and microvascular disease [62]. It has been observed that the lower the glycaemic control, the higher the risk of developing periodontitis [63]. The pathogenic processes linking the two diseases are not well defined, but it is believed that the over-regulated inflammation resulting from each condition adversely affects the other. Indeed, in diabetic patients, there is increased deposition of advanced glycation end products (AGEs) in periodontal tissues, and interactions between AGEs and their receptor on macrophages (RAGE) lead to activation of the local immune system, resulting in increased cytokine secretion, oxidative stress and bone resorption [64]. In contrast, the reverse relationship, or the impact of periodontitis on diabetes, is explained by the production and release of inflammatory cytokines and other mediators produced locally in inflamed periodontal tissues following bacterial stimulation, passing into the circulation and contributing to over-regulated systemic inflammation. This leads to impaired insulin signalling and insulin resistance, resulting in the exacerbation of diabetes [65]. Therefore, emerging evidence suggests that the interrelationship between diabetes mellitus and periodontal disease, based on the intracellular level, reflects a vicious circle among oxidative stress and inflammation [66,67]. Epigenetics could be one of the main players in developing multifactorial diseases such as diabetes mellitus and periodontal disease, and miRNAs could act as epigenetic regulators [68]. miRNA-146a and miRNA-155, which are co-induced in many cell types responding to microbial lipopolysaccharide, facilitate negative feedback control of NFκB target genes encoding various mediators of inflammation [69] and are involved in oxidative stress by targeting superoxide dismutase (SOD) [70]. Overexpression of miRNA146a and miRNA155 was found in the crevicular fluid of diabetic and non-diabetic patients with chronic periodontitis. These patients underwent non-surgical therapy, and in both groups, regardless of the diabetes factor, a decrease in the expression of these miRNAs was observed. Furthermore, at baseline, it was seen that miRNA146a expression levels were higher in the crevicular fluid of diabetic patients with periodontitis than in patients with periodontitis alone [60]. The levels of miRNA-223, miRNA-203 and miRNA-200b were assessed in the gingival crevicular fluid and serum of patients with chronic periodontitis, chronic periodontitis and type 2 diabetes and healthy controls. The study indicated a significant increase in miRNA-223 expression associated with a significant positive correlation with TNF-α and clinical parameters in the chronic periodontitis groups with and without diabetes compared to healthy controls, associating miRNA-223 with inflammation, neutrophil recruitment and the pathogenesis of chronic periodontitis [71]. In the study conducted by Chen, it was indicated that TNF-α participates in the pathogenesis of periodontitis [72]. A significantly lower expression of miR-203 was found in chronic periodontitis groups with and without diabetes compared to healthy controls. Therefore, significantly lower expression of miR-203 in the patient groups and a significant negative correlation with TNF-α explain decreased healing, suggesting its impact on irreversible damage caused by the disease [71]. MiRNA-200b showed overexpression in both serum and gingival crevicular fluid of both patient groups with respect a control group [71], results that agree with other works [39,73]. In contrast, several studies have shown dysregulation of miR-223 in type 2 diabetes, induced by advanced glycation end products, which consequently defines apoptosis of osteoblasts and endothelial cells in diabetes mellitus [53]. Furthermore, it has been reported that some miRNAs, including miR-223, have been deregulated years prior to the manifestation of type 2 diabetes [74].

Obesity, a chronic inflammatory condition, has a major impact on the pathophysiology of periodontal disease. Indeed, several studies have reported that obesity is associated with a higher prevalence and severity of periodontal disease [75,76]. This relationship has been associated with the exposure of macrophages to a hyperlipidaemic environment, which alters their functional capacities, including decreased cytokine production [77,78]. In a study, differences in the miRNA expression profiles of gingival tissue in periodontitis when obesity was present were evaluated, and miRNA microarray profiling of gingival tissue samples revealed differential miRNA expression in obese individuals with periodontitis compared to normal-weight participants. Among the 13 miRNAs upregulated and 22 miRNAs downregulated in the presence of obesity, the expression of miR-200b-5p was 1.6-fold. Gingival tissue biopsies from patients with periodontal disease also showed an inverse correlation between miR-200b-5p expression and the mRNA expression of its target genes, namely ZEB1, ZEB2, GATA2 and KDR. In detail, ZEB2 and GATA2 were statistically significant and ZEB1 and KDR were close to significance [71]. These genes play a central role in pathways involved in the re-epithelialisation of gingival wounds, an important process in periodontal regeneration and restoration of tissue integrity, which affects treatment outcome [79]. Upregulation of miRNA-200b/c alters TLR4 signalling in macrophages, affecting innate host defences against periodontal pathogens [40], while miR-200c-5p was found to be increased in patients with inflammatory bowel disease [80]. There are no other studies reporting miR-200b expression in the gingival tissue of obesity patients. In experimental models of obesity, diet-induced liver damage was correlated with increased levels of miRNA-200b in mouse plasma [81] and in mouse and rat liver tissue [82]. One of the known factors that regulates the hepatic expression of miRNA-200b [83] and the regenerative capacity of the periodontium [60] is the metabolic hormone leptin. The hormone leptin negatively interferes with periodontal ligament cells’ regenerative capacity, suggesting leptin as a pathomechanical link between obesity and impaired periodontal healing [84]. Angiogenesis plays a key role in periodontitis by facilitating the transport of oxygen and nutrients to the site of injury and the removal of cellular debris from inflamed tissue [85]. Under-regulation of endothelial miR-200b induces over-regulation of the activity of the transcription factor GATA2. This regulates the promoters of many endothelial genes and thus positively regulates angiogenetic activity [86]. In obese individuals, upregulation of miR-200b results in decreased expression of GATA2 and KDR, both of which play a central role in the angiogenic response and wound healing.

Table 2 presents a summary of the miRNAs implicated in periodontal disease and related to other systemic diseases, heart disease, obesity and diabetes. It briefly summarises the above.

## 4. Exosomes and Periodontal Disease

**Figure 2:**
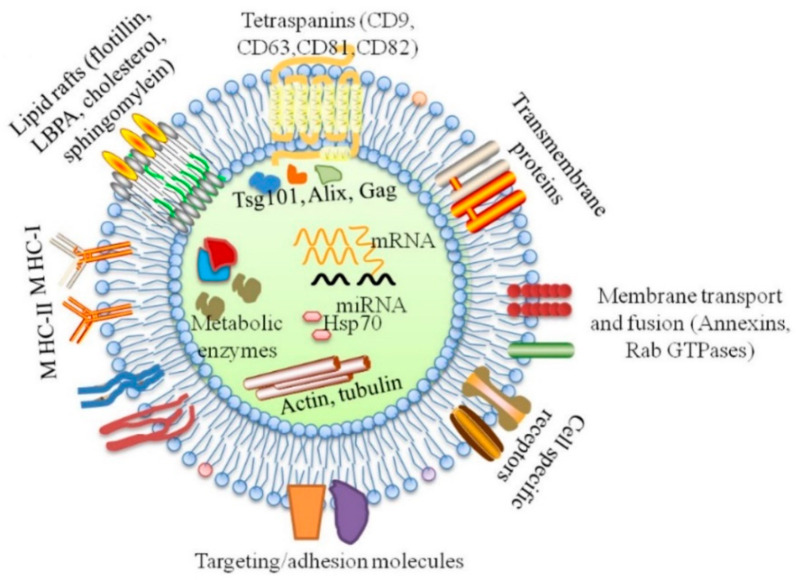
Main constituents of molecules included in exosomes. Many proteins are common among all exosomes regardless of their maternal cell types, including tetraspanins, fotillin, heat shock proteins (HSP70, HSP90), MHC I, GTPases (Rab, RAL) and endosome-associated proteins (Alix, Tsg101). Exosomes are also enriched in lipid rafts on their surfaces, including fotillin, LBPA, cholesterol, sphingomylein and nucleic acids in the lumen, including DNAs (mtDNA, ssDNA, dsDNA) and RNAs (mRNA, miRNA, rRNA and tRNA) [19].

**Figure 3:**
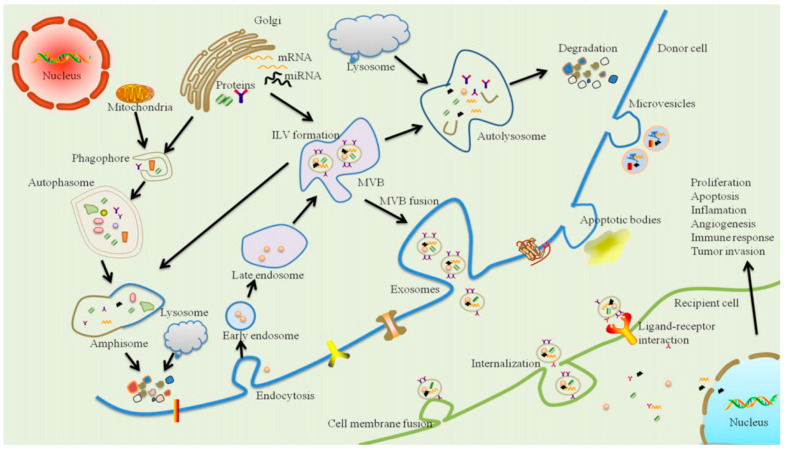
Schematic representation of exosome biogenesis, release and intercellular communication. Exosomes originate from an endocytic compartment. The inward budding of plasma membrane forms an early endosome. During maturation of the early endosome, the inward budding of limited areas of the endosomal membrane to form intraluminal vesicles (ILVs) produces multivesicular bodies (MVBs). MVBs face two fates, where some of them are delivered to lysosomes or autophasomes for degradation, while others fuse with the plasma membrane, inducing the secretion of exosomes. During the inward process of ILVs, many cytoplasmic components are encapsulated, such as proteins, lipids and nucleic materials, so they represent a new signalling paradigm to interfere with cell-to-cell communication. Moreover, this intercellular signal transmission might be mediated through three pathways, including endocytosis/internalisation, direct membrane fusion and receptor–ligand interaction [19].

Exosomes are lipid vesicles enriched in specific miRNAs and other molecules (Figure 2). It is known that exosomes play the role of intracellular and extracellular transporters. Successful isolation of exosomes has been demonstrated by the detection of the exosome markers, tetraspanins, CD9, CD63, Hsp70 and CD81. Tetraspanins can interact with integrins, mediating cell adhesion to the extracellular matrix and facilitating intracellular transport (Figure 3). [87]. In recent years, exosomes have come to represent a new signalling paradigm to mediate intercellular communication because of their capacity to exchange components, including proteins, nucleic acids and lipids [88,89]. In recent years, the role of exosomes in tumour development has been much discussed. Tumour-derived exosomes are of great interest as they are implicated in the promotion of tumour proliferation, migration capacity, invasion capacity and immune suppression in the tumour microenvironment [90]. Exosomes have been shown to play a role in the regulation of inflammatory and immune diseases, such as rheumatoid arthritis, Sjogren’s syndrome and systemic lupus erythematosus [91]. Only recently has research been showing interest in the possible pathogenetic relationship between periodontitis and exosomes, as recent evidence has highlighted their potential in the diagnostic and therapeutic fields [19].

Periodontal ligament fibroblasts (PDLFs) are the main cell population involved in the early stages of periodontal inflammation [92]. After stimulation with lipopolysaccharide (LPS), exosomes derived from human PDLFs slightly increase the expression of IL-6 and TNF-α in osteoblasts and, at the same time, significantly inhibit the expression of osteogenesis-related elements (including collagen-I and osteoprotegerin) by reducing alkaline phosphatase activity [93]. Compared to exosomes extracted from periodontal ligament stem cells normal (PDLSCs), exosomes derived from LPS-stimulated PDLSCs have a higher amount of miRNA-155 and its downstream target Sirtuin-1. This results in reduced expression of T-helper cells 17 (Th17) and increased expression of Treg (regulatory T cells), reducing inflammation through the Th17/Treg/miRNA-155-5p/Sirtuin-1 regulatory network [94]. A recent publication on miRNAs as a salivary biomarker conducted by Fujimori’s group indicated that out of 84 miRNAs profiled, only hsa-miRNA-381-3p showed a significant difference between patients without periodontitis or with mild periodontitis compared to the group with severe periodontitis. Confirming the above results, quantitative real-time polymerase reaction (qPCR) showed no significant differences [95]. In a subsequent study, exosomal miRNA profiles were evaluated as possible biomarkers for chronic periodontitis by analysing eight plasma and eight salivary–exosomal miRNA samples. The results showed that plasma and salivary exosomes are a reliable subject for miRNA studies. In fact, the miRNA profile detected in plasma and serum exosomes and their expression is concordant with the profile of miRNAs found in tissue and crevicular fluid in various studies in the literature [28,96]. Furthermore, it appears that miRNA-let-7d, miRNA-126-3p and miRNA-199a -3p, in exosomal plasma samples, may be reliable candidates for the development of possible biomarkers of periodontitis, whereas miR-125a-3p could be a possible biomarker of periodontitis in lysosomal saliva samples. In conclusion, these miRNAs are reliable candidates for the development of periodontitis biomarkers as there were significant differences in their expression, with good discriminatory value and a strong correlation with the mean value of periodontal pocket depth (PPD) [97]. 

In recent years, the potential of using exosomes in bone and periodontal regeneration has been demonstrated. In fact, the use of exosomes has been tested in the diagnosis of early-stage lung cancer [98] and in several other diseases as a diagnostic tool [99]. Exosomes can also be isolated in saliva, leading to significant advantages due to its non-invasive nature, no risk of bleeding, good patient compliance, plasma-like content and ease of collection [100]. However, only a limited amount of clinical data are currently available. The use of exosomes has been investigated in organs after ischemia–reperfusion injury in animal models, and exosomes have been shown to provide potent cardio protection [101]. This can be attributed to the heat shock protein HSP-70 and the activation of the toll-like receptor TLR-4. It has been shown that in skin regeneration, exosomes have the ability to modulate each stage of healing through the delivery of various molecules, such as mRNA and miRNA, trophic factors and functional proteins [102]. Several studies have been conducted on the role of exosomes in bone and periodontal regeneration. They indicated an improvement in bone and periodontal regeneration, determined by the activation of the *Wnt/β-catenin* pathway, which induces angiogenesis/vasculogenesis [103]. Furthermore, it has been indicated that lysosomes induce the promotion of cell migration and proliferation, playing a role in tissue regeneration [104]. Thus, exosomes, according to Wang et al., may have pro- or anti-inflammatory effects, depending on the component transported [105]. In order to improve the usefulness of exosome applications in healing outcomes, future studies should focus on exosome sources and conditions. Several studies have shown that exosome therapy appears to improve bone and periodontal regeneration significantly. A dose-related effect has also been observed; however, no study has compared the influence of the source of the exosomes on the promotion of tissue healing and bone/periodontal regeneration, as revealed by a recent systematic review [106].

## 5. Clinical Potential and Future Perspective in Periodontal Disease 

Multiple studies on miRNAs have been conducted to find possible biomarkers that can be used in the diagnosis of periodontal disease [107]. Initially, studies were conducted on gingival tissue, but, recently, interest has shifted to gingival crevicular fluid and saliva as possible sources of miRNAs [108]. The reason that biofluids have been proposed as excellent research sources for candidate miRNA biomarkers are as follows: the ease of isolation and identification of miRNAs by quantitative polymerase chain reaction (qPCR); the less invasive nature of collecting miRNA samples (saliva and gingival crevicular fluid provide an opportunity for non-invasive testing); and the high stability of miRNAs in various biofluids commonly used in analytical assays [109,110]. Several miRNAs have been proposed as future biomarkers for PD. Among the most relevant is miRNA-21-3p, which is related to the MAPK tumour signalling pathway, T-lymphocyte receptors, adhesion molecules, etc. [33]. MiRNA-146 in association with miRNA-155 is a key regulator of the immune system, promoting the expression of certain cytokines, such as tumour necrosis factor-alpha (TNF α), IL-1 β, type I and type II, interferons (IFN) or RANKL. The expression of both miRNAs is implicated in chronic inflammation [111]. Finally, miRNA-200 is related to the mesenchymal–epithelial transition by regulating the expression of the transcription factor ZEB-1 [112]. Currently, it is difficult to clinically determine a group of miRNAs as biomarkers for periodontal disease, as the studies performed represent only first phase of their study. Therefore, further studies are required.

Individual miRNAs can be used as therapeutic agents. They can target several genes and influence multiple regulatory networks. Furthermore, a combination of miRNAs and their antagonists can be used to regulate different members of the same signalling pathway. For example, to limit bone resorption, some miRNAs could be used to restore the function of other lost or under expressed miRNAs related to osteoblastogenesis or vice versa to inhibit the function of other upregulated miRNAs related to osteoclast differentiation and function. More than a dozen miRNA delivery systems have been developed in recent years, mainly divided into viral and non-viral. Viral miRNA delivery systems rely on the action of retroviruses, lentiviruses and adenoviruses; however, these systems induce a strong immune response, which could reduce their effectiveness [113,114,115]. On the other hand, non-viral approaches face the difficult challenge of having to transport miRNAs, or some of their antagonists, across the cell membrane, preserving their structure en route to the nucleus [115]. These non-viral miRNA delivery approaches include lipid-based systems (such as liposomes) and polymer-based approaches, including polyethylenimine (PEI), poly(lactic-co-glycolic acid) (PLGA) and poly(amidoamine) (PAMAM). Other recently developed miRNA carriers include gold, iron and silica-based nanoparticles [115,116,117,118], as well as chitosan, protamine and collagen [115]. Munagala et al. used exosomes to encapsulate chemotherapeutic and chemopreventive agents against lung cancer. They demonstrated, in vivo, that functionalised exosomes have an anti-tumour effect [118]. In another study, curcumin, combined with peptides, was placed inside exosomes and it was shown to have a greater anti-inflammatory effect than conventional exosomes [119]. Therefore, the use of exosomes seems to be a future therapy with potential clinical application in the treatment of periodontal defects. Recently, it was observed that periodontal regeneration was mediated by mesenchymal stem cells (MSCs), through the secretion of exosomes. In fact, collagen sponges containing human MSCs-Exos were used to treat periodontal intra-bony defects in rats. The results showed that intra-bony defects healed more efficiently than in control rats with newly formed bone and periodontal ligament (PDL). Subsequent experiments conducted on PDL cells showed that human MSC-Exos are rapidly taken up by PDL cells, promoting the migration and proliferation of PDL cells. This occurs due to CD73 adenosine receptor-mediated activation of the AKT and ERK signalling pathways [120]. Furthermore, it has been observed that exosomes secreted by adipose-derived stem cells (ASCs-Exos) have a better therapeutic effect on ligature-induced periodontitis than ADSCs themselves, as they result in a greater area of newly formed tissue [121].

## 6. Conclusions

The literature shows that miRNAs have high diagnostic, prognostic and therapeutic potential, although we still need to refine our knowledge of their activity in periodontal disease. However, the results are very encouraging, especially those aimed at finding possible miRNAs as biomarkers in saliva and gingival crevicular fluid, as their collection is non-invasive and easy to perform. Therefore, the diagnostic, prognostic and therapeutic methods of tissue, salivary and gingival crevicular fluid miRNAs seem feasible, but standardised and precisely followed criteria and protocols need to be established to obtain comparable and reproducible results. In the future, exosomes could be excellent biomarkers due to their ability to accurately reflect disease pathogenesis and their easy and wide availability in different types of body fluids (such as crevicular fluid and saliva) and in body cells and tissues. As diagnostic and prognostic biomarkers of periodontal disease, they may have enormous potential, but this is still an under-explored area that requires further investigation. Studies investigating the role of miRNAs and exosomes as possible therapeutic agents are also promising. Considerable results have been achieved in tissue regeneration. However, their use as therapeutic agents still needs to be defined and investigated.

## Figures and Tables

**Table 1:** Summary of relevant miRNAs in periodontal disease.

miRNAs	miRNA in Diseased Tissues	Functions	Reference
miRNA-548	Upregulation	Upregulation of IL-8 within the periodontal pocket	[26,27]
miRNA-31	Upregulation	Regulates the expression of ICAM-1, which controls the migration of leukocytes from the bloodstream to the tissues	[29]
miRNA-17	Upregulation	Regulates the expression of E- Selectin, which controls the migration of leukocytes from the bloodstream to the tissues	[15]
miRNA146	Upregulation	Negatively regulates the TLR signalling pathway	[30,31]
miRNA-146a	Upregulation	Negatively regulates TLR signalling; reduced expression of NF-κb, TNFα, IL-1β and IL-6, which induce osteoclastogenesis	[54]
miRNA-146b	Upregulation	Negatively regulates TLR signalling	[54]
miRNA-155	Downregulation	Regulates TLR release in inflamed tissues	[54]
miRNA-200	Upregulation	Reduces the release of IL-6, IL-8, IFRD1 and NF-κb	[38]
miRNA-200c	Upregulation	Regulatory effect on TLR4-mediated signalling in macrophages	[39]
miRNA-21	Upregulation	Decreases NF-κb activation	[41]
miRNA-let-7	Upregulation	Inhibits TLR4	[42]
miRNA-203	Downregulation	Promotes neo-angiogenesis and regulates innate immunity	[10,28]
miRNA-223	Upregulation	Plays a role in alveolar bone loss	[48,50]

**Table 2:** miRNA profiles in periodontal disease and systemic disease.

mRNAs	Correlation with Systemic Disease	Activity	Expression	References
mRNA-146a	Heart disease	Chronic inflammatory disorders, both cardiac and periodontal diseases. Acts against Th1 and Th2 cells, shifting the balance towards Th1.	Upregulation in tissues of patient with periodontal and heart diseases	[30]
mRNA-let7	Heart disease	Inhibition of angiogenesis through up-regulation of TSP-1.	Upregulation in tissues of patient with periodontal and heart diseases.	[43,45]
mRNA-146	Diabetes	Negative feedback control of NFκB target genes and are involved in oxidative stress by targeting SOD.	Overexpression in crevicular fluid of diabetic patients with periodontitis.	[60]
mRNA-155	Diabetes	Negative feedback control of NFκB target genes and are involved in oxidative stress by targeting SOD.	Overexpression in crevicular fluid of diabetic patients with periodontitis.	[60]
mRNA-223	Diabetes	Increased expression of TNF-α.	Upregulation in tissue, crevicular fluid and serum in patients with diabetes and periodontitis.	[71]
mRNA-203	Diabetes	Reduced expression of TNF-α.	Downregulation in tissue, crevicular fluid and serum in patients with diabetes and periodontitis	[71]
mRNA-200-3p	Diabetes	Increased expression of TNF-α.	Upregulation in tissue, crevicular fluid and serum in patients with diabetes and periodontitis.	[71]
mRNA-200b-5p	Obesity	Reduced expression of its target genes ZEB1, ZEB2, GATA2 and KDR involved in re-epithelisation.	Upregulation in tissue of obese patients with periodontitis.	[73]
mRNA-200b/c	Obesity	Alters TLR4 signalling in macrophages.	Upregulation in tissue of obese patients with periodontitis.	[40]
